# Anatomical Study of the Superficial Musculoaponeurotic System in Relation to the Zygomaticus Major

**DOI:** 10.3390/diagnostics14182066

**Published:** 2024-09-18

**Authors:** Hyun-Jin Park, Mi-Sun Hur

**Affiliations:** Department of Anatomy, Daegu Catholic University School of Medicine, Daegu 42472, Republic of Korea; hjpark321@cu.ac.kr

**Keywords:** superficial musculo-aponeurotic system, zygomaticus major, orbicularis oculi

## Abstract

Background: The superficial musculoaponeurotic system (SMAS) is crucial for the structural integrity and dynamics of facial expressions and is a particularly important consideration during facelift surgeries. This study investigated the anatomical structure and continuity of the SMAS at the site where the zygomaticus major (Zmj) originates, which is where the SMAS extends from the lateral to the anterior aspects of the face. Knowledge of these aspects is crucial for understanding the mechanics of facial movements and also the aging process. Methods: Dissections of 66 specimens and histological analyses were used to explore the intricate relationships and attachments between the SMAS and facial muscles. Results: The findings indicated that at the Zmj origin site, the SMAS—connected to the inferior margin of the orbicularis oculi—covered the superficial surface of the Zmj fibers. As it tracked downward, the SMAS was observed to split into two layers lateral to the Zmj fibers, enveloping them both superficially and deeply. Additionally, as the SMAS continued forward, it ceased to be distinctly visible in the buccal area. Conclusions: These results provide a deeper understanding of the complex layering and interconnectivity of the SMAS, which supports facial dynamics and structural integrity. This information could be particularly useful in surgical and aesthetic procedures in the midfacial area.

## 1. Introduction

Facelift surgeries have evolved from simple skin-tightening techniques to more complex approaches that include the superficial musculoaponeurotic system (SMAS). The initial methods applied for skin tightening produced only minor changes, but the introduction of the SMAS concept by Mitz and Peyronie in the 1970s led to a new era of targeting deeper tissue layers in facial rejuvenation [1]. Various SMAS techniques such as plication and lifting have subsequently become integral to facelift procedures, effectively addressing midlife facial aging to produce more natural results. Advanced SMAS lifting techniques now consider the entire structure connected to facial muscles, and they yield more refined outcomes while preserving natural beauty and minimizing the signs of aging [2,3]. Additionally, the SMAS flap construction technique is frequently employed in facelift surgeries, reconstruction of the parotid lodge, and parotidectomy, further emphasizing its critical role in various facial surgical procedures [4,5,6].

SMAS lifting and flap-construction procedures consider the anatomical relationship between the SMAS and the zygomaticus major (Zmj) [7,8]. The vector direction in which SMAS is redraped in facelift surgeries is often described in relation to the Zmj, so that suspension prevents distortion of the mimetic musculature and a “facelifted appearance” [9].

The SMAS forms a continuous layer from the frontalis through the orbicularis oculi (OOc) and Zmj to the platysma along the side of the face. While the frontalis, OOc, and platysma are not attached to bone and create a continuous layer with the SMAS, the Zmj originates from bone and so has difficulty forming a continuous layer structure with the SMAS. Therefore, the anatomical structure and continuity of the SMAS are of particular interest in areas where muscles originate from bone and where the OOc and Zmj overlap. Few detailed studies of these specific anatomical relationships have been reported in the literature.

The histology of the SMAS has been investigated extensively across various facial areas, particularly that of the relationship between the SMAS and facial creases. The histological classification into Type I and Type II SMAS by Ghassemi et al. (2003) has been crucial in understanding these relationships [10]. Furthermore, Sandulescu et al. (2015) detailed the anatomical nuances and surgical implications of SMAS interactions with the Zmj, particularly emphasizing the significance of SMAS positioning and its impact on facial aesthetics in surgical procedures [11]. These classifications and findings are crucial to understanding the structural transitions at the boundary of the Zmj origin, which is a key area as the SMAS extends toward the anterior aspect of the face.

The purpose of this study was to characterize the anatomical structure and continuity of the SMAS as they relate to facelift surgeries, particularly focusing on areas where muscles such as the OOc and Zmj overlap and where muscles originate from bone. Given the rarity of detailed descriptions in the literature and the complexity of these specific anatomical relationships, our research aimed to provide a clearer understanding of the SMAS, which is essential for improving the efficacy and outcomes of facelift procedures.

## 2. Materials and Methods

### 2.1. Specimens and Dissection of Cadavers

The SMAS adjacent to the Zmj was examined in 66 specimens from 34 embalmed adult Korean cadavers (17 males and 17 females), donated to the Catholic Kwandong University College of Medicine, with a mean age at death of 74.9 years (age range 40–99 years). There was no history of midface trauma or surgery. In 62 of the 66 specimens, the skin, subcutaneous tissue, and facial muscles were dissected to expose the SMAS, Zmj, and OOc. At the Zmj origin site, the continuity and positional relationships of the layers of the SMAS, Zmj, and OOc were examined. The attachment of the SMAS to the Zmj was also observed, tracking down along the Zmj. The remaining four specimens were reserved for histological analysis to provide a detailed assessment of the microstructural configurations. The study was exempted by the Institutional Review Board (IRB) of Catholic Kwandong University (IRB number CKU-21-01-1103). The study was performed in accordance with the Declaration of Helsinki arising from the 64th WMA General Assembly in Fortaleza, Brazil in October 2013.

### 2.2. Staining for Histological Analyses

Histological analyses were performed on four specimens stained using Masson’s trichrome. The Zmj specimens were initially cut into three parts. These parts were further sectioned at 5 mm intervals in the transverse plane, starting from the origin of the Zmj fibers and moving downward along their course. Histological evaluations of the continuity and positional relationships of the layers of the SMAS, Zmj, and OOc were performed by staining 5 μm thick transverse sections near the Zmj origin site.

### 2.3. Ultrasonographic Image Analyses

Ultrasonographic images were obtained using a real-time two-dimensional B-mode ultrasound device featuring a high-frequency (18 MHz) linear transducer (Sonimage HS1; KONICA MINOLTA, Tokyo, Japan). Nontoxic ultrasonography gel (Meditop Sono Jelly, Meditop, Seoul, Republic of Korea) was applied during the ultrasonographic image scanning process. Ultrasonographic image scanning was performed at the origin of the Zmj and vertical line passing through the lateral canthus. 

## 3. Results

### 3.1. Gross Anatomy of Positional Relationships of the SMAS, Zmj, and OOc

**Figure 1:**
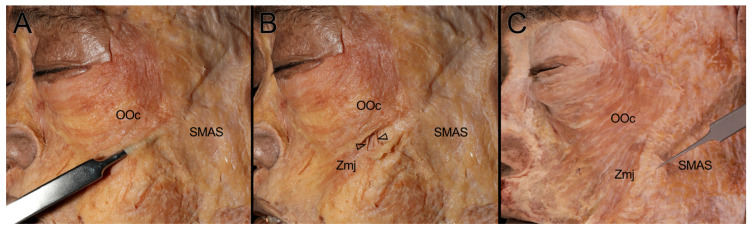
Layered anatomical relationship of the Zmj, OOc, and SMAS (**A**) The SMAS covered the arising fibers of the Zmj and was attached to the lateral margin of the inferolateral border of the OOc. A pair of forceps were inserted through the SMAS below the inferolateral margin of the OOc from the superficial surface of the Zmj to reveal that the SMAS covered the arising fibers of the Zmj. (**B**) The SMAS, which covered the arising fibers (arrowheads) of the Zmj, was incised at the inferolateral margin of the OOc and retracted superiorly to expose these Zmj fibers. (**C**) The SMAS covered the arising fibers of the Zmj and was attached to the lateral margin of the upper fibers of the Zmj, approximately up to the vertical line passing through the lateral canthus. A pair of forceps were used to retract the SMAS attached to the lateral border of the Zmj.

In all observed specimens, the inferior margin of the OOc covered the arising fibers of the Zmj at its origin site. The SMAS was connected to the inferolateral margin of the OOc, and also partially covered the arising fibers of the Zmj. Cutting the SMAS attached to the inferolateral margin of the OOc exposed the arising fibers of the Zmj. The lateral margin of the Zmj was attached to the SMAS down to approximately the vertical line passing through the lateral canthus. From this vertical line inward, corresponding to the distal one-third of the Zmj, the SMAS transitioned into a loose connective tissue structure, where it reattached to the lateral margin of the Zmj (Figure 1).

**Figure 2:**
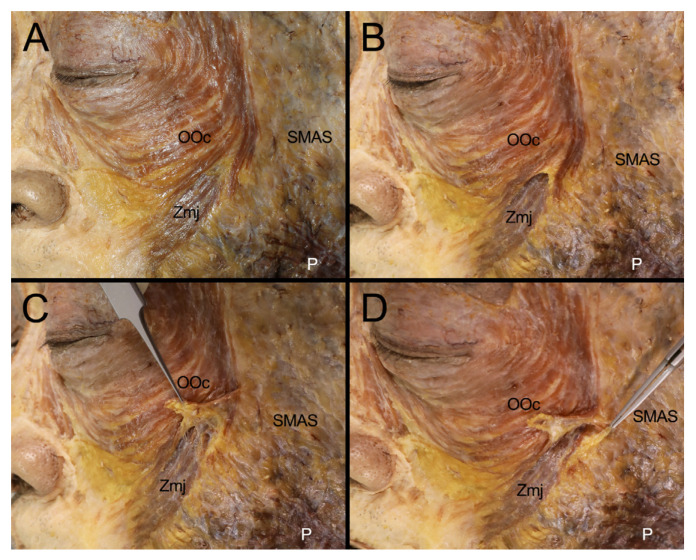
Attachments of the SMAS in multiple layers to the Zmj and OOc (**A**) The arising fibers of the Zmj at its origin were covered by the inferolateral margin of the OOc. (**B**) Connective tissues and fat were removed to clearly reveal the border of the inferolateral margin of the OOc and the lateral fibers of the malaris, which were located superficially to the Zmj arising fibers at its origin site. (**C**) The inferolateral margin of the OOc and lateral fibers of the malaris at the origin of the Zmj were reflected superiorly to expose the arising fibers of the Zmj. (**D**) The SMAS was attached to both the lateral border of the upper fibers of the Zmj and the inferolateral margin of the OOc. P, platysma.

In addition, the dissection further elucidated the complex attachments of the SMAS in multiple layers to both the Zmj and OOc. It was ultimately observed that the SMAS was simultaneously attached to both the lateral border of the upper fibers of the Zmj and the inferolateral margin of the OOc, indicating the intricate and layered connections of these facial structures (Figure 2).

### 3.2. Analyzes of the Sectioned Images and the Corresponding Histological Images

**Figure 3:**
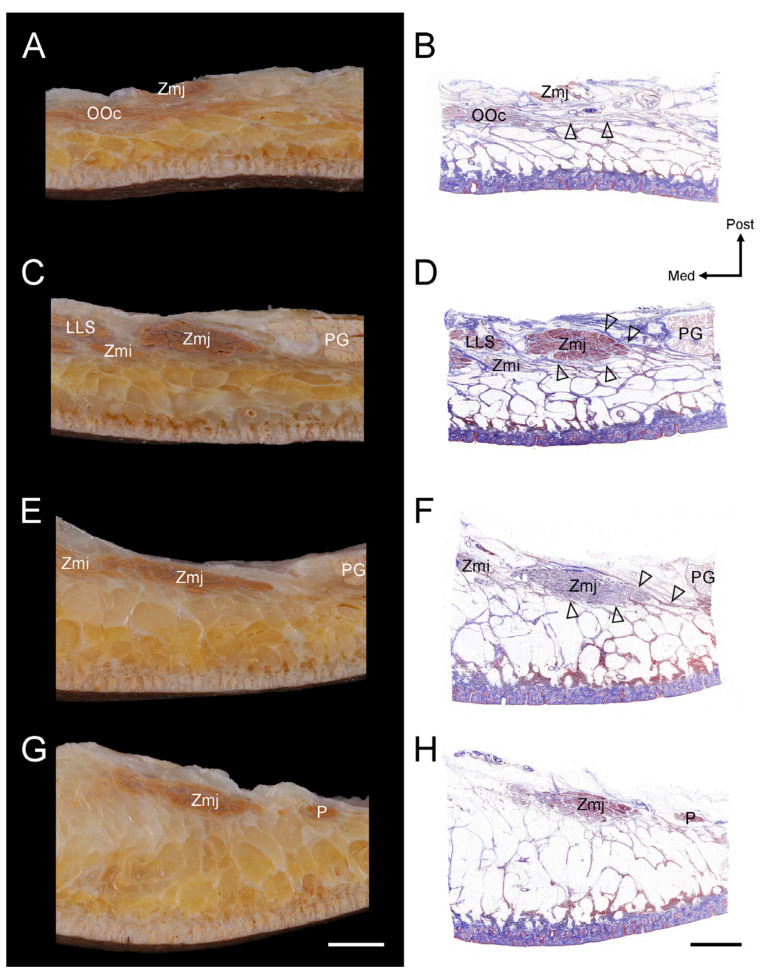
Histological images of the layered relationships of the SMAS with the Zmj and OOc Transverse histological sections along the course of the Zmj detail its interactions with the SMAS and OOc. (**A**,**B**) At the Zmj origin, the SMAS connected to the inferior margin of the OOc and covered the superficial surface of the arising fibers of the Zmj (arrowheads). (**C**,**D**) Just lateral to the Zmj fibers, the SMAS divided into two layers that enveloped the Zmj fibers both superficially and deeply (arrowheads). The SMAS merged with the parotid fascia adjacent to the parotid gland. (**E**,**F**) At the level of the middle fibers, as the Zmj fibers extended and flattened, the surrounding SMAS similarly became thinner (arrowheads). (**G**,**H**) The SMAS was not distinctly visible near the insertion point of the Zmj. All sections were viewed at a magnification of x200. Scale bars indicate 0.5 cm. LLS, levator labii superioris; Med, medial; Post, posterior; PG, parotid gland; P, platysma; Zmi, zygomaticus minor.

1. Transverse sectioning was performed starting from the origin of the Zmj fibers, progressing downward along their course. The following observations were made at various levels.
2. At the Zmj origin site, the SMAS connecting to the inferior margin of the OOc covered the superficial surface of the arising fibers of the Zmj at its origin (Figure 3A,B).
3. At the level of the arising fibers of the Zmj, the SMAS divided into two layers just lateral to the Zmj fibers, which then enveloped the Zmj fibers both superficially and deeply. The SMAS blended with the parotid fascia on the parotid gland (Figure 3C,D).
4. At the level of the middle fibers of the Zmj, the Zmj fibers became flattened, and the SMAS enveloping these fibers correspondingly became thinner (Figure 3E,F).
5. At the level of the distal fibers of the Zmj before its insertion, the SMAS was no longer distinctly visible (Figure 3G,H).

### 3.3. Ultrasound Images of the Layered Relationship of the SMAS with Zmj and OOc 

The horizontal and longitudinal lines passing through the Zmj origin could be used as references for defining the layered relationships and attachment patterns of the SMAS, Zmj, and OOc. In the longitudinal view, the relationships between OOc, Zmj, and SMAS could be observed, starting from the inferior fibers of the OOc and moving downward. The arising fibers of the Zmj at its origin were covered by the SMAS extending downward from the OOc and connected from the lateral side. 

**Figure 4:**
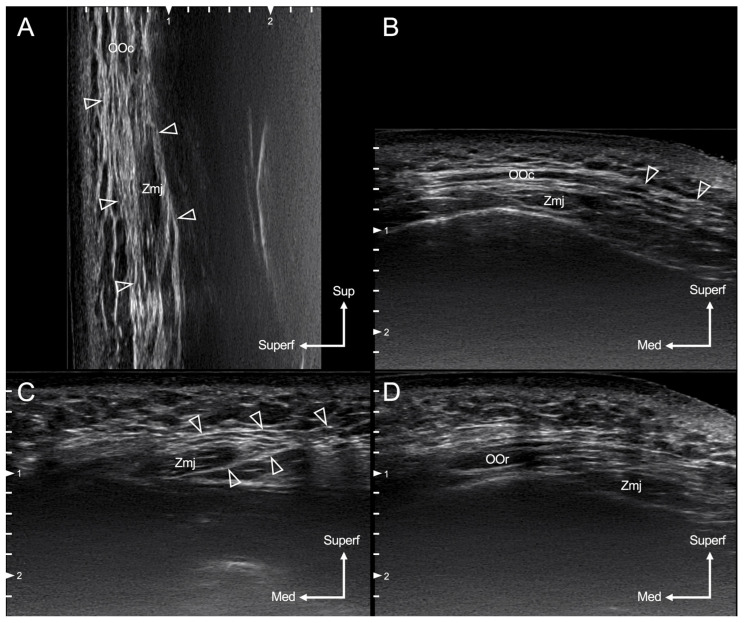
Ultrasound images of the layered relationships of the SMAS with the Zmj and OOc. The arising fibers of the Zmj at its origin were covered by the OOc and SMAS, with the SMAS extending (arrowheads) downward from the OOc, as observed in both longitudinal (**A**) and transverse (**B**) views. (**C**) Lateral to the vertical line passing through the lateral canthus, the Zmj fibers were enveloped by the SMAS both superficially and deeply (arrowheads), with the SMAS being thicker on the surface. (**D**) Prior to inserting into the orbicularis oris (OOr), the Zmj fibers were not clearly attached to the SMAS medial to the vertical line passing through the lateral canthus. Med, medial; Sup, superior; Superf, superficial.

In the transverse view, the layered relationships of the Zmj, SMAS, and OOc could be observed along the course of the Zmj fibers medial from the lateral side on the face. The attachment patterns of the SMAS and Zmj were observed while moving medially along a vertical line passing through the lateral canthus. The Zmj fibers were enveloped by the SMAS both superficially and deeply lateral to this vertical line, with the SMAS being thicker on the surface. Prior to the Zmj fibers inserting into the orbicularis oris, the Zmj fibers were not clearly attached to the SMAS medial to this vertical line (Figure 4).

## 4. Discussion

The SMAS was connected to the inferolateral margin of the OOc, covering the arising fibers of the Zmj from its origin site. As the Zmj fibers descended, the SMAS was attached to the lateral margin of the upper fibers of the Zmj. Therefore, in the upper region of Zmj, the SMAS was interconnected with the OOc and Zmj according to its respective levels. 

Positional changes of the SMAS are thought to occur because this musculoaponeurotic system that is attached to the OOc covers the deeply overlapping arising fibers of the Zmj and then reattaches to the upper fibers of the Zmj due to the overlap between the OOc and Zmj. The SMAS attaches to the OOc in the upper face, to the upper fibers of the Zmj (excluding the arising fibers at its origin site) in the midface, and to the platysma in the lower face. This arrangement connects the muscles around the forehead, orbit, zygomatic regions, and lateral parts of the cheeks to facilitate muscle movements and transmit tension across these regions.

Standring (2020) stated that the SMAS becomes indistinct on the lateral aspect of the face approximately 1 cm below the level of the zygomatic arch and blends with the epimysia of certain mimetic muscles, including the Zmj [12]. Additionally, where the SMAS overlies the parotid gland, it blends firmly with the superficial layer of the parotid fascia. In the present study, it was observed that the attachment of the SMAS to the Zmj became indistinct around the vertical line passing through the lateral canthus. This finding indicates the variability in SMAS attachments and its transition to a more-diffuse connective tissue structure in certain regions.

De la Cuadra-Blanco et al. (2013) observed that in certain development stages, the SMAS does not exhibit continuity with the Zmj [13]. They further noted the absence of structures analogous to the SMAS in the labial region, underscoring their distinct developmental trajectories. Conversely, Barton (1992) reported that as the SMAS approached the nasolabial fold, it divided into superficial and deep fascial leaves to envelop the Zmj and the zygomaticus minor [14]. This division, combined with the anchoring of the SMAS at the bony origin of the Zmj in the zygomatic arch, impairs the transmission of tension from the cheek flap to the skin of the nasolabial fold. 

Ghassemi et al. (2003) performed detailed histological analysis to reveal that the SMAS in the zygomatic, infraorbital, parotid, and lateral parts of the nasolabial fold region comprises a network of both vertical and horizontal fibrous septa [10]. These septa create structured fat lobules that provide the essential dynamic support for facial expressions. Anchored to the periosteum of the zygomatic bone and directly attached to the Zmj, this configuration facilitates movements that are critical to smiling. Therefore, maximizing the tension in the skin during facelift procedures requires the severing of the attachments of the SMAS to the zygomatic muscles. In the present study, the SMAS covered the arising fibers of the Zmj from its origin site and was attached to the lateral margin of the upper fibers of the Zmj. This attachment implies the important role of the connection between the SMAS and Zmj in facial procedures, emphasizing the need to consider these attachments for ensuring optimal surgical outcomes.

Furthermore, Park et al. (2024) classified the positional relationships of the Zmj origin with the nasal ala and the tragus into three categories and found that a horizontal line through the center of the Zmj origin and the nasal ala passed through the tragus in 41.7% of their specimens [15]. This anatomical relationship indicates that both the variability and complexity of the relationship between the SMAS and Zmj must be carefully considered when planning surgical procedures.

This study also used ultrasound to observe the attachments of the SMAS to surrounding structures (including the Zmj) and their variations, as well as to identify and track the changes in the positions of these structures on a layer-by-layer basis. The ultrasound findings from this study will make it easier for surgeons to examine and verify the anatomical structures of each individual before performing a surgical procedure, thereby facilitating the planning of various procedures related to SMAS lifting and flap construction.

This study has provided significant insights into the layered anatomical configuration and interactive dynamics underlying facial expressions, which have potential implications for surgical interventions in the periorbital region. Detailed knowledge of the SMAS connections may enhance our understanding of the intricate and complex features of facial anatomy. This information is important to consider when performing surgical and aesthetic procedures involving the midface and may aid predictions of the anatomical behaviors of the SMAS and related structures.

## Data Availability

The data presented in this study are available on request from the corresponding author.
